# Kaleidoscopic Views in the Bone Marrow: Oxalate Crystals in a Patient Presenting with Bicytopenia

**DOI:** 10.4274/tjh.2015.0256

**Published:** 2016-02-17

**Authors:** Yelda Dere, Simge Erbil, Murat Sezak, Başak Doğanavşargil, Mümtaz Yılmaz, Nazan Özsan, Mine Hekimgil

**Affiliations:** 1 Sıtkı Koçman University Faculty of Medicine, Department of Pathology, Muğla, Turkey; 2 Ege University Faculty of Medicine, Department of Pathology, İzmir, Turkey; 3 Ege University Faculty of Medicine, Department of Nephrology, İzmir, Turkey

**Keywords:** Oxalosis, Hyperoxaluria, Bone marrow

## TO THE EDITOR

**Figure 1:**
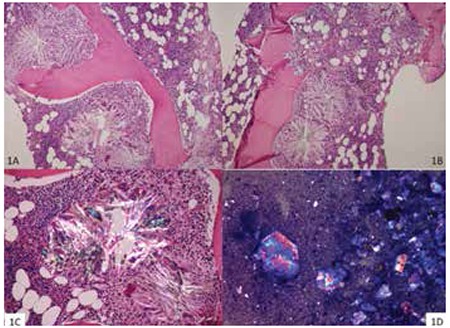
A, B: Characteristic appearance of oxalate crystals in the bone marrow, H&E, 100x. C: Colorful rosette-like crystal under polarized light, H&E, 200x. D: Colorful envelope-like crystal in the bone marrow aspirate, Giemsa stain, 100x.

Our patient is a 24-year-old female who was admitted to the nephrology clinic of our hospital with fatigue, weakness, and swelling of the feet. From her medical history, we learned that she had two operations for nephrolithiasis at the ages of 9 and 12, and she underwent renal transplantation in 2013, but she was still on hemodialysis. Laboratory tests showed bicytopenia (anemia and leukopenia) with hemoglobin of 8.2 g/dL and white blood cell count of 3800/mm3, and she underwent a bone marrow (BM) biopsy. Microscopically, an almost complete suppression of hematopoietic cells with the replacement of BM cells by foreign-body reactive fibrous tissue and numerous birefringent crystalline materials were detected (Figures 1A and 1B). The crystals formed rosettes with needle-like radial extensions surrounded by foreign body-type giant cells. Under polarized light, the crystals formed multicolored rosettes (Figure 1C). In BM aspirates envelope-like crystals were found in the background of a few myeloid cells with normal morphology (Figure 1D). Based on histopathological examination integrated with clinical results, a diagnosis of hypocellular BM associated with crystal deposition concordant with oxalate crystals was made. Because of the absence of genetic tests performed to date, the patient was referred to the genetics department, and after genetic studies the diagnosis of primary hyperoxaluria was confirmed.

Pancytopenia associated with BM infiltration of different deposits is a rare condition mostly associated with amyloidosis or the accumulation of iron. One of the rarest deposits in the BM is oxalate crystals due to hyperoxaluria [1,2,3]. Primary hyperoxaluria, a genetic disorder due to mutation in the alanine glyoxylate aminotransferase gene, located on chromosome 2q37.3 and resulting in the conversion of glyoxylate to oxalate, is characterized by increased production of oxalic acid because of the specific liver enzyme deficiency and generally presents with renal stones, renal or liver failure, and oxalosis [4]. Calcium oxalate may even be deposited into various tissues such as those of the retina, peripheral nerves, arterial media, and heart [4,5]. The medical history of nephrolithiasis at early ages, characteristic appearance of birefringent crystals forming rosettes in the BM, and the envelope-like forms in the BM aspirates seen in our case supported the diagnosis of primary hyperoxaluria, which is best confirmed by genetic studies and treated with liver transplantation because of the location of the abnormal enzymes in the hepatocytes.
